# Short-term EGFR blockade enhances immune-mediated cytotoxicity of EGFR mutant lung cancer cells: rationale for combination therapies

**DOI:** 10.1038/cddis.2016.297

**Published:** 2016-09-29

**Authors:** Charli Dominguez, Kwong-Yok Tsang, Claudia Palena

**Affiliations:** 1 Laboratory of Tumor Immunology and Biology, Center for Cancer Research, National Cancer Institute, National Institutes of Health, Bethesda, MD, USA

## Abstract

The epidermal growth factor receptor tyrosine kinase inhibitor (EGFR-TKI) erlotinib has been approved for years as a first-line therapy for patients harboring EGFR-sensitizing mutations. With the promising implementation of immunotherapeutic strategies for the treatment of lung cancer, there is a growing interest in developing combinatorial therapies that could utilize immune approaches in the context of conventional or targeted therapies. Tumor cells are known to evade immune attack by multiple strategies, including undergoing phenotypic plasticity via a process designated as the epithelial–mesenchymal transition (EMT). As signaling through EGFR is a major inducer of EMT in epithelial cells, we have investigated the effect of EGFR inhibition with erlotinib on tumor phenotype and susceptibility to immune attack. Our data shows that short-term exposure of tumor cells to low-dose erlotinib modulates tumor plasticity and immune-mediated cytotoxicity in lung cancer cells harboring a sensitizing EGFR mutation, leading to a remarkable enhancement of tumor lysis mediated by innate NK cells and antigen-specific T cells. This effect positively correlated with the ability of short-term EGFR blockade to modulate tumor phenotype towards a more epithelial one, as well as to increase susceptibility to caspase-mediated apoptosis. The effect, however, was lost when erlotinib was utilized for long periods of time *in vitro* or *in vivo*, which resulted in gain of mesenchymal features and decreased (rather than increased) tumor lysis in response to immune effector mechanisms. Our data provides rationale for potential combinations of erlotinib and immunotherapies for the treatment of lung carcinomas in the early setting, before the establishment of tumor relapse with long-term EGFR inhibition.

Lung cancer is the leading cause of cancer related deaths in the world, with the majority of cases (~85%) corresponding to the non-small cell lung cancer (NSCLC) type.^1^ Frequently, proliferation and survival of NSCLC is driven by the oncogene epidermal growth factor receptor (EGFR), which results deregulated in tumors by means of mutation or gene amplification.^2^ The frequency of EGFR deregulation in NSCLC has led to the development of several EGFR-targeted therapies, including erlotinib, an approved EGFR tyrosine kinase inhibitor (EGFR-TKIs) that is widely used for the treatment of NSCLC.^3, 4^ Presence of EGFR-sensitizing mutations, mainly deletions in exon 19 or the L858R substitution in exon 21 of EGFR, has shown to best predict responses to erlotinib and other EGFR-TKIs in patients;^5^ however, despite a remarkable tumor debulking caused by EGFR signaling blockade, tumor relapse is seen in almost 100% of treated patients, resulting in very low long-term survival rates.^6^

Given the high mortality rate of NSCLC, there is a pressing call for novel therapeutic approaches that could circumvent the therapeutic resistance seen in the clinic thus far.^7^ In recent years, immunotherapeutic strategies have become recognized means to stimulate the destruction of tumor cells by manipulating or enhancing anti-tumor immune responses. However, there is still a need for further improvement of immune-mediated approaches for the treatment of NSCLC.^8^ One aspect for consideration in this regard is the ability of tumor cells to evade immune responses by an array of strategies,^9, 10^ including their ability to undergo profound phenotypic plasticity via a process designated as the epithelial–mesenchymal transition (EMT).^11, 12^ This phenomenon is now recognized as a mechanism of progression in carcinomas,^13, 14, 15^ where it promotes tumor cell migration, invasion and metastasis, as well as the acquisition of resistance to a variety of anti-cancer therapeutics, including resistance to immune-mediated cytotoxic lysis.^16, 17, 18, 19^

Accumulating evidence links the EGF/EGFR axis to the phenotypic plasticity of solid tumors. In multiple reports in different cancer types, signaling through this axis has been described as a driver of tumor EMT^20, 21^ and given these observations, blockade of EGFR signaling has been explored and shown to revert the phenotype of tumor cells from a mesenchymal-like to an epithelial one.^20, 22^ Interestingly, long exposure to EGFR-TKIs, resulting in the acquisition of tumor resistance and relapse, has been linked to the acquisition of mesenchymal properties in tumor cells, as mesenchymal-like cells are less sensitive to the cytotoxic effects of EGFR inhibition.^23, 24, 25, 26^

Although the effect of EGFR-TKIs in cancer signaling and clinical outcome are well established, the potential of blocking EGFR signaling as a means to facilitate tumor targeting by anti-tumor immune effector cells has not been thoroughly investigated. In this study, EGFR inhibition has been explored both *in vitro* and *in vivo* with xenografts of EGFR-mutated NSCLC cells, in terms of its ability to modulate epithelial *versus* mesenchymal features and to improve tumor sensitivity to immune-mediated attack. Our data demonstrate that short-term, low-dose erlotinib modulates immune-mediated cytotoxicity of NSCLC cells, leading to a remarkable enhancement of tumor cell lysis. This effect positively correlated with the ability of short-term blockade of EGFR signaling to modulate tumor phenotype towards a more epithelial one. The effect, however, was lost when erlotinib was utilized for long periods of time (⩾72 h both *in vitro* or *in vivo*), which resulted in tumor mesenchymalization and decreased tumor lysis in response to immune effector mechanisms. The data presented here thus provide rationale for potential combinations of erlotinib and immunotherapies for the treatment of lung carcinomas in the early setting, before the establishment of tumor relapse with long-term treatment with an EGFR-TKI.

## Results

### Erlotinib induces time-dependent phenotypic alterations in NSCLC cells

**Figure 1:**
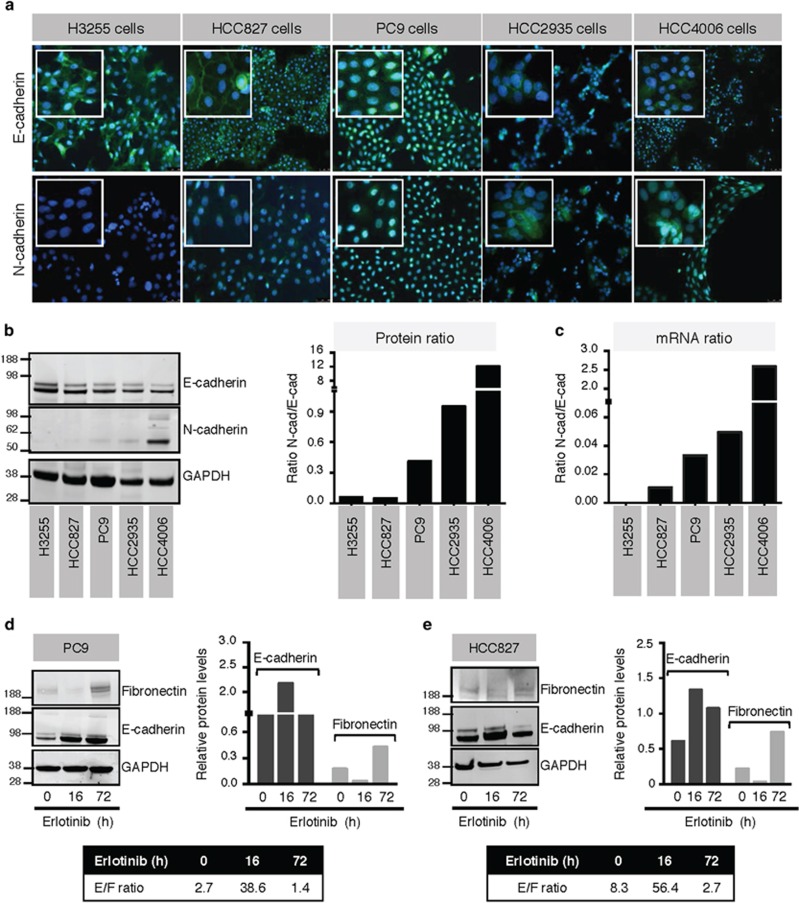
Mutated NSCLC cell lines display varying EMT phenotypes. (**a**) Immunofluorescent and (**b**) western blot analysis of E-cadherin and N-cadherin expression in five mutated NSCLC cell lines. The ratio of N-cadherin: E-cadherin is also shown at the protein (**b**) and mRNA (**c**) levels. PC9 (**d**) and HCC827 (**e**) cells were treated with erlotinib for indicated times; lysates were evaluated via western blot for E-cadherin and fibronectin and quantified. Shown in the bar graph is the expression of each protein relative to GAPDH; the box shows the ratio of E-cadherin: fibronectin expression at each time point. Original magnification of all images: 20 × ; blue corresponds to 4′,6-diamidino-2-phenylindole (DAPI)-stained nuclei

Five NSCLC cell lines harboring EGFR-sensitizing mutations were chosen to characterize their phenotypic response to erlotinib. The levels of epithelial E-cadherin and mesenchymal N-cadherin expression were assessed by immunofluorescence (Figure 1a), western blot (Figure 1b), and polymerase chain reaction (PCR) (Figure 1c). Although the cell lines harbor similar EGFR mutations, a range of phenotypes was observed among the various lines, with H3255 and HCC827 cells exhibiting an epithelial phenotype, whereas an intermediate status characterized by variable co-expression of epithelial and mesenchymal cadherins could be observed in PC9, HCC2935 and HCC4006 cells. Analysis of EGFR expression levels in the five cell lines revealed no major differences, with the exception of slightly lower levels in HCC4006 cells (Supplementary Figure 1A). Previously, work from our laboratory and others have indicated that long-term exposure to erlotinib can induce resistance in NSCLC cells due to the acquisition of a mesenchymal-like status.^27^ To assess whether erlotinib could lead to phenotypic modulation of NSCLC cells in a time-dependent fashion, HCC827 and PC9 cells were exposed to erlotinib for 16 *versus* 72 h. As shown in Figures 1d and e, 16-h treatment with erlotinib induced a marked increase of E-cadherin and a substantial decrease of fibronectin expression resulting in a marked increase in E-cadherin/fibronectin (E/F) ratio, indicating that short-term blockade of EGFR signaling could be effective at reducing mesenchymal NSCLC traits. The effect, however, was lost when tumor cells were pre-treated with erlotinib (72 h). There was a remarkable overexpression of mesenchymal fibronectin with a resulting low E/F ratio for both cell lines, compared with the 16-h treatment. These observations were substantiated by immunofluorescence analysis of HCC827 cells (Supplementary Figure 1B). Given these data, we concluded that rapid time-dependent changes in phenotype could be achieved after erlotinib treatment of EGFR-mutated lung cancer cell lines.

### Rapid tumor phenotypic changes induced by erlotinib *in vivo*

**Figure 2:**
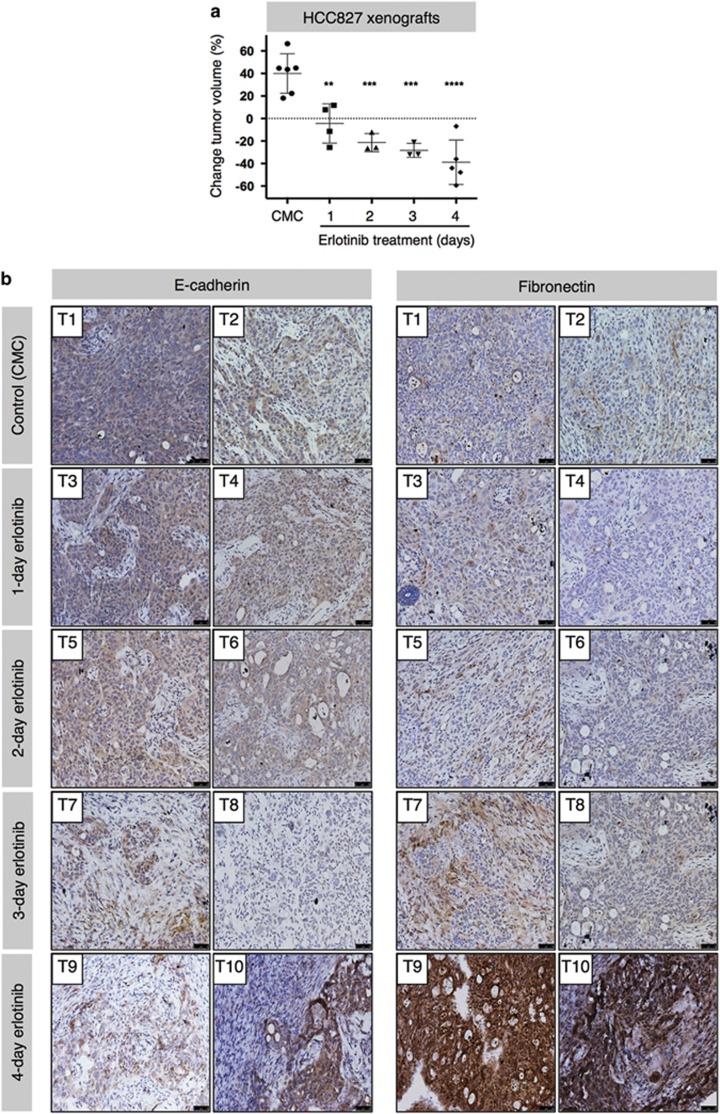
Erlotinib treatment induces mesenchymalization *in vivo*. (**a**) Mice with HCC827 xenografts were treated with carboxymethyl cellulose control or erlotinib for indicated times; tumor volume change was assessed. (**b**) Tumor tissue sections were stained for E-cadherin and fibronectin to evaluate protein expression via IHC. Tissues were counterstained with hematoxylin. Original magnification of all images: 20 ×. Error bars depict S.D. of sample size (*n*=4–5) from each treatment group

To evaluate if the rapid phenotypic modulation seen *in vitro* could also be relevant *in vivo*, HCC827 and HCC4006 cells were grown as xenografts in immune-deficient mice receiving 1–4 injections of erlotinib. As predicted, there was a significant dose-dependent reduction in HCC827 tumor volumes (Figure 2a). Remarkably, considerable changes in EMT markers were observed as well (Figure 2b). It became evident that a 4-day erlotinib administration *in vivo* was able to induce a mesenchymal-like phenotype, as apparent by a marked increase in fibronectin expression seen with immunohistochemistry (IHC, Figure 2b, lower panels). This phenomenon was also seen with HCC4006 xenografts, where a reduction in tumor volume and a more mesenchymal phenotype were observed after 4-day treatment (Supplementary Figures 2A and B). These data for the first time highlighted the ability of erlotinib to rapidly induce EMT features *in vivo*.

### Erlotinib induces time-dependent changes in immune-mediated lysis

**Figure 3:**
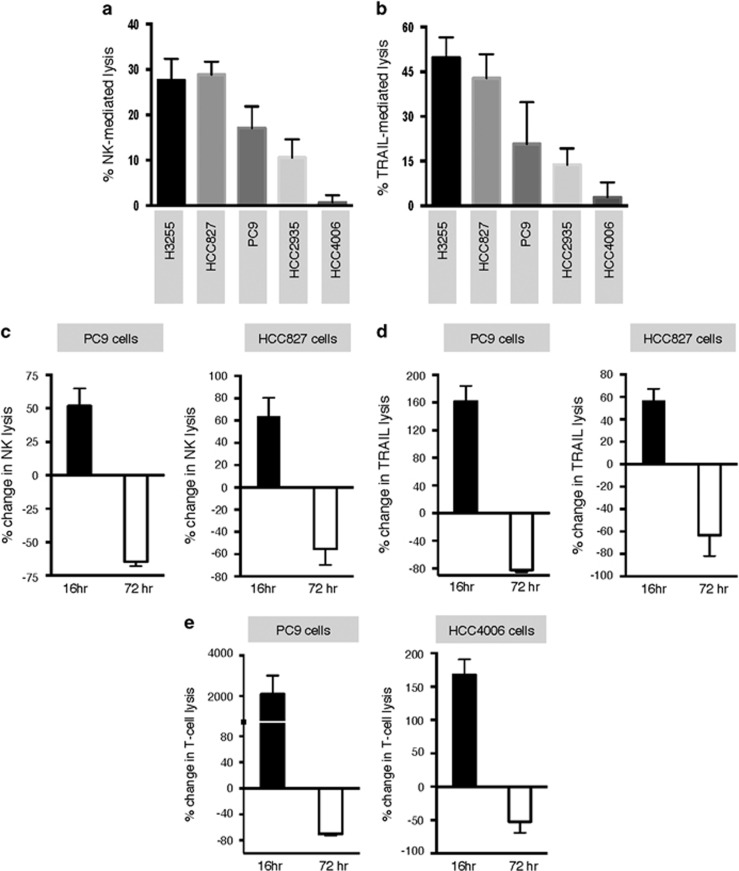
Erlotinib induces a time-dependent susceptibility to immune lysis in NSCLC cells. Susceptibility to immune-mediated lysis was simultaneously assessed in all five NSCLC lines utilizing (**a**) NK effector cells from a single donor at 25:1 ratio, or (**b**) 250 ng/ml TRAIL. Lysis of PC9 and HCC827 cells mediated by (**c**) NK effector cells or (**d**) TRAIL, with erlotinib directly added to the 16-h cytotoxic assay or used for 72-h treatment of tumor cells before the cytotoxic assay. Shown is the % change of lysis observed for erlotinib-treated *versus* control untreated tumor cells. (**e**) Susceptibility of PC9 and HCC4006 cells treated with erlotinib (16 *versus* 72 h) *versus* control untreated cells, using brachyury-specific (left panel) or MUC1-specific T cells (right panel) as effectors, respectively

In previous studies, our group and others have shown that acquisition of mesenchymal traits by carcinoma cells could lead to resistance to immune-mediated cytotoxicity.^17, 18, 28^ Consistent with those reports, lung cancer cells with a more epithelial phenotype (H3255, HCC827) demonstrated a higher susceptibility to natural killer (NK) cells (Figure 3a) or TRAIL-mediated lysis (Figure 3b), compared with cells with a more mesenchymal phenotype (HCC4006). We then investigated the ability of erlotinib to modulate tumor responses to immune lysis. For these experiments, tumor cells were pre-treated with erlotinib for 72 h and exposed to immune effector cells or recombinant TNF-related apoptosis-inducing ligand (TRAIL), or in contrast, tumor cells were exposed to immune effector cells or TRAIL in the presence of erlotinib (16-h assay). Strikingly, 16-h erlotinib markedly increased tumor lysis by NK cells (Figure 3c) or TRAIL (Figure 3d) in PC9 and HCC827 cells, whereas 72-h pre-treatment proved detrimental in both PC9 and HCC827 cells. This time-dependent change in tumor susceptibility to lysis was corroborated using brachyury-specific or MUC1-specific T cells with PC9 and HCC4006 cells, respectively (Figure 3e).

### Short-term erlotinib treatment modulates apoptotic threshold of tumor cells

**Figure 4:**
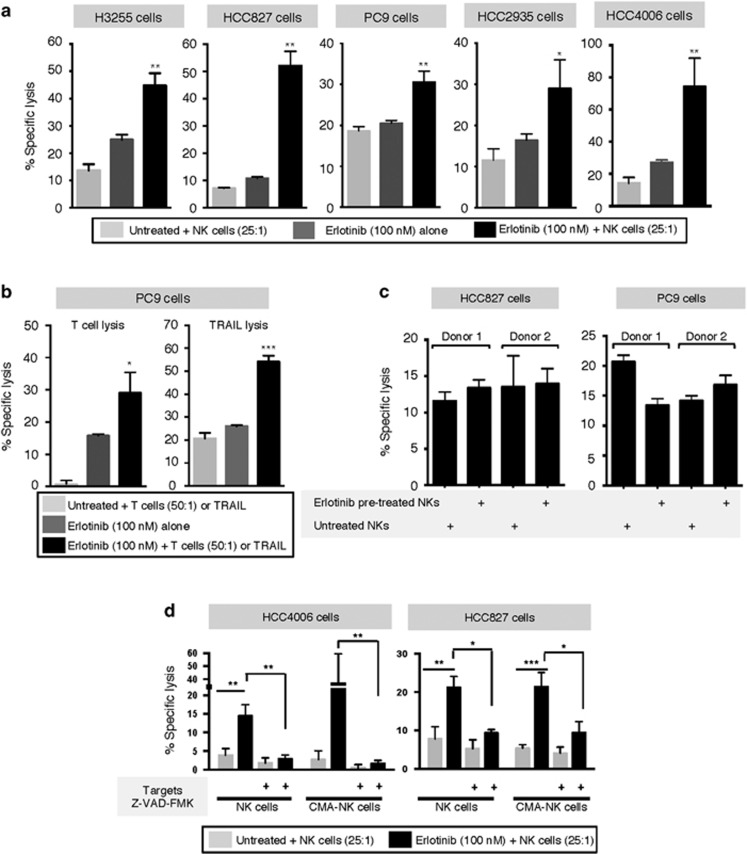
Enhancement of lysis is caspase-dependent. (**a**) Lysis of indicated tumor cell lines mediated by NK cells alone, erlotinib alone, or NK cells in the presence of erlotinib (16-h assay). (**b**) Susceptibility to lysis by brachyury-specific T cells and TRAIL (500 ng/ml), with or without simultaneous erlotinib treatment in PC9 cells. (**c**) Specific lysis of HCC827 and PC9 cells with isolated NK cells pre-treated with erlotinib for 16 h before the cytotoxic assay or left untreated. (**d**) NK-mediated lysis of HCC4006 and HCC827 cells that were untreated or pretreated with Z-VAD-FMK; effector NK cells were untreated or pre-treated with CMA. As indicated, the assay was conducted with or without erlotinib

The effect of simultaneous erlotinib treatment was further evaluated with all five cell lines. As shown in Figure 4a, simultaneous erlotinib significantly enhanced the lysis of all cell lines in response to effector NK cells, when compared with the lysis mediated by NK cells or erlotinib alone. Similar results were observed with brachyury-specific T cells or TRAIL in PC9 cells (Figure 4b), where simultaneous erlotinib administration significantly enhanced tumor lysis above the level observed with each treatment alone.

To investigate the mechanism responsible for the enhanced response to immune attack, we began by analyzing whether erlotinib could directly enhance the effector function of immune cells. Isolated NK cells were exposed to erlotinib for 16 h and used as effectors for lysis of tumor cells in comparison to untreated NK cells. As shown in Figure 4c, lysis of either HCC827 or PC9 cells with erlotinib pre-treated NK cells was not improved when compared with the lysis observed with untreated NK cells. It was then hypothesized that short-term erlotinib treatment may induce a general improvement of apoptosis in target cells. Utilizing caspase and granzyme/perforin blockade, the mechanism of lysis was investigated. The effect of simultaneous erlotinib treatment was completely abrogated when HCC4006 and HCC827 targets were pre-treated with the pan-caspase inhibitor Z-VAD-FMK (Figure 4d), indicating a role for caspase-dependent cytotoxicity. Our results also demonstrated that perforin/granzyme pathways had no contribution towards the enhancement of lysis observed in the presence of erlotinib, as the pre-treatment of NK effector cells with the granzyme/perforin inhibitor Concanamycin A (CMA) was unable to prevent the effects of erlotinib.

**Figure 5:**
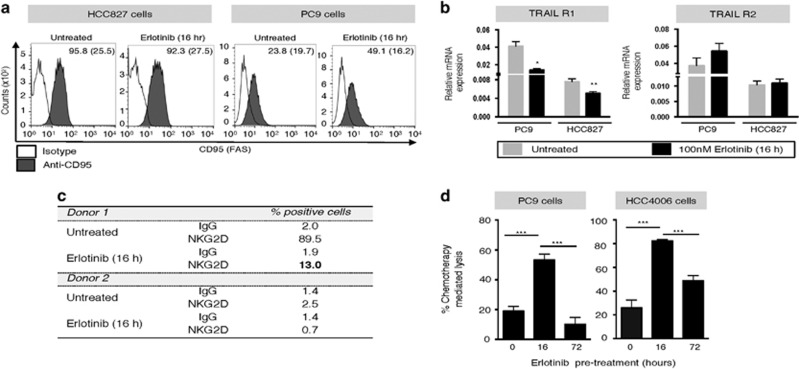
Erlotinib effect on surface-associated death receptors and other immune-relevant molecules. (**a**) FAS expression levels were measured via FACS analysis in HCC827 and PC9 cells. (**b**) TRAIL receptor-1 and -2 mRNA levels were measured via qRT-PCR in cells treated as indicated. (**c**) NKG2D receptor levels were assessed via FACS analysis on NK cells isolated from two normal donors. (**d**) PC9 or HCC4006 cells were left untreated (0 h) or were pre-treated with 100 nM erlotinib for 16 or 72 h. Tumor cells were collected, washed and subsequently exposed to a mixture of the chemotherapeutics cisplatin and vinorelbine for 96 h, or left untreated. Cell survival was evaluated by Cell Titer-Glo; represented is the percentage of tumor cell lysis induced by chemotherapy treatment, calculated for each group (erlotinib 0, 16, or 72 h) as: [fluorescent counts chemotherapy treated wells/fluorescent counts untreated wells] × 100

To investigate the potential role of FAS and TRAIL receptor upregulation in the above observations, their expression was evaluated in tumor cells exposed to erlotinib for 16 h. Although no change was observed in FAS expression on HCC827 cells, an increase was seen with PC9 cells (Figure 5a). TRAIL receptors-1 and -2 were also assessed via mRNA expression, observing a decrease or no changes in both cell lines (Figure 5b). These data ruled out upregulation of surface-associated death receptors as the mechanism for enhanced tumor lysis in the presence of erlotinib. In previous reports, however, EGFR signaling has been shown to modulate expression of immune-relevant molecules in tumor cells, including modulation of MHC class I signaling via STAT1/SHP2 pathways^29^ and transcriptional regulation of MHC class I.^30^ Our results showed a decrease rather than an increase on the expression of the NK-activating receptor natural killer group 2, member D (NKG2D) on the surface of NK cells from two different donors, following 16-h erlotinib treatment (Figure 5c). In addition, no changes in MHC Class I, ICAM-1, LFA-3, B7-1 or MHC class I polypeptide-related sequence A (MICA) were observed (data not shown). Subsequent studies with PC9 and HCC4006 cells exposed to erlotinib for 16 *versus* 72 h and subsequently treated with cisplatin and vinorelbine also demonstrated that short-term erlotinib can significantly enhance the cytotoxic activity of chemotherapy (Figure 5d). These results indicated that the effect of short-term erlotinib may be due to a general improvement of apoptosis in target cells.

### Cells resistant to cytotoxicity express stem cell and anti-apoptotic markers

**Figure 6:**
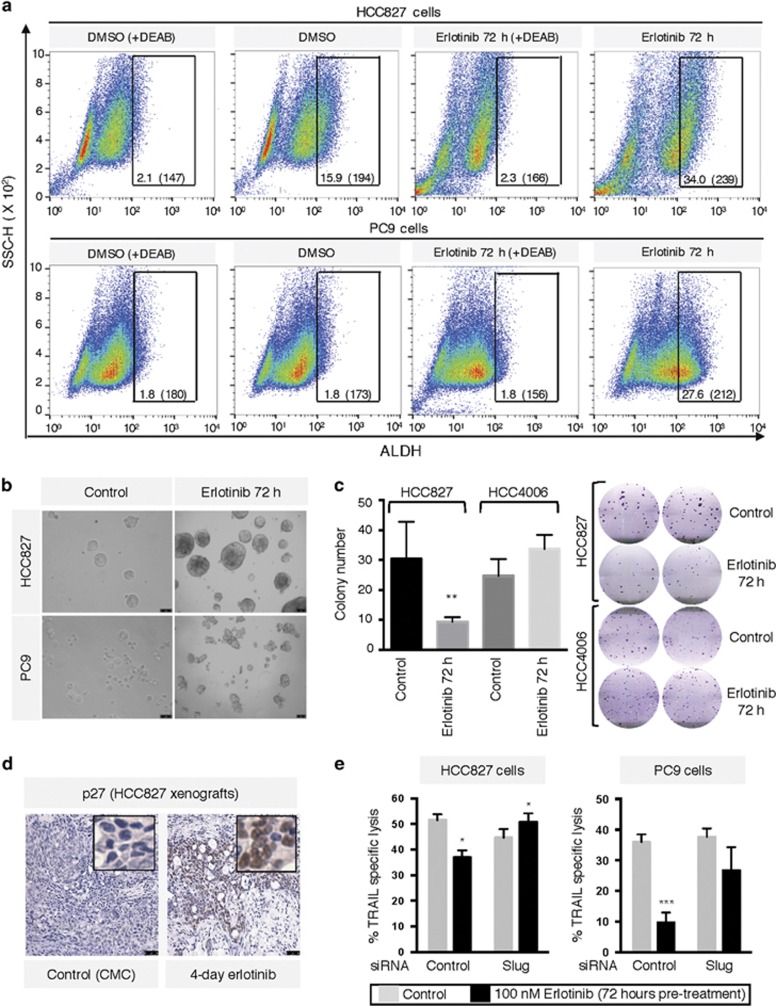
Pre-treatment with erlotinib induces stem cell-like features. (**a**) Staining of tumor cells for expression of ALDH following pre-treatment with erlotinib via FACS. Numbers indicate the percentage of ALDH^*bright*^ cells and the corresponding mean fluorescence intensity. (**b**) Tumor spheroid growth was assessed in both cells lines as indicated in ‘Materials and Methods' section. (**c**) Colony formation assay with indicated tumor cell lines left untreated or pre-treated with 100 nM erlotinib for 72 h. Graph depicts the average colony number from triplicate wells; right panel shows representative wells for each cell line. (**d**) HCC827 xenograft sections were stained for p27 via IHC. Tissues were counterstained with hematoxylin; original magnification of all images: 20 ×. (**e**) HCC827 and PC9 cells were transfected with a control non-targeting or a pool of small interfering RNAs directed against Slug; each transfected group was left untreated or exposed to erlotinib for 72 h and subsequently utilized in a TRAIL-mediated assay

Cancer stem-like cells (CSCs) have been found in many cancer types and have been implicated in aggressive disease course and resistance to treatment.^31^ Tumors harboring CSCs have an increased propensity for metastasis, recurrence, and often respond inadequately to standard care therapies.^32^ As the mesenchymal phenotype has been increasingly linked to CSCs,^33^ we evaluated their presence among cell populations that no longer responded to immune-mediated lysis following 72-h erlotinib. Analysis of the CSC marker aldehyde dehydrogenase (ALDH)^34^ demonstrated a marked increase in the fraction of ALDH^+^ cells in HCC827 and PC9 cells exposed to erlotinib for 72 h (Figure 6a). Pluripotency and self-renewal features were also investigated by evaluating the ability of the tumor cells to give rise to spheroids in culture, observing increased number of spheroids formed by cells that were exposed to erlotinib for 72 h before culture, compared with control cells (Figure 6b). A colony formation assay performed after 72-h erlotinib treatment also showed significantly decreased colony formation with epithelial HCC827 but not mesenchymal HCC4006 cells (Figure 6c). Tumor lysates from untreated *versus* erlotinib pre-treated HCC827 and PC9 cells were also analyzed using multi-protein arrays for expression of apoptosis-related proteins. These experiments revealed a remarkable increase in p27 expression in HCC827 and PC9 cells following 3-day erlotinib treatment (Supplementary Figure 3A). The p27 protein has been previously implicated in resistance to multiple therapies via its inhibitory effect on apoptosis^35^ and has also been linked to cell proliferation and poor prognosis.^36^ In addition, upregulated p27 expression was confirmed *in vivo* with HCC827 xenografts following 4-day erlotinib treatment (Figure 6d). Lastly, as the phenomenon of EMT and CSCs appear to be linked, we conducted studies to analyze whether known drivers of EMT could play a role in the resistance seen after 72-h erlotinib. Substantial upregulation of Slug mRNA, a transcription factor previously implicated in resistance to apoptosis^37^ was observed following erlotinib treatment for 3 days (Supplementary Figure 3B). When tumor cells were silenced for expression of Slug simultaneously to their exposure to erlotinib for 72 h, a successful reconstitution of TRAIL-mediated lysis was achieved with HCC827 and PC9 cells (Figure 6e).

### Erlotinib pre-treatment selects for mesenchymal cells resistant to cytotoxicity

**Figure 7:**
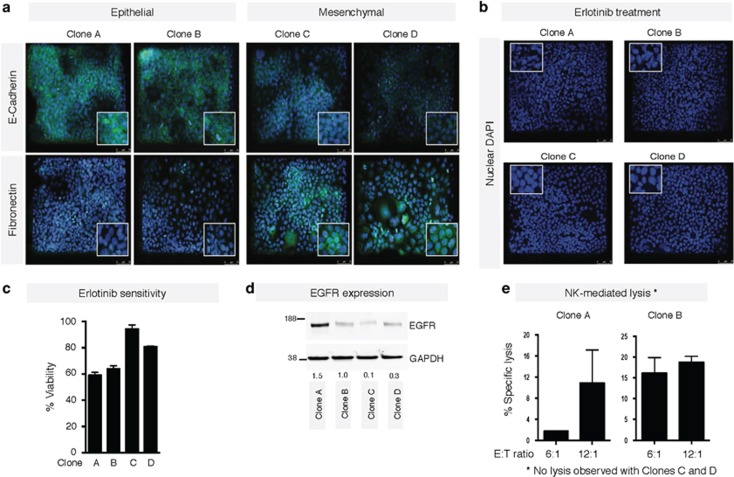
Selection of mesenchymal cells by pre-treatment with erlotinib. (**a**) Immunofluorescence of HCC827 clones for epithelial E-cadherin and mesenchymal fibronectin. After erlotinib treatment, erlotinib sensitivity was evaluated via (**b**) immunofluorescence for DAPI nuclear staining, or (**c**) cell viability using Cell Titer-Glo. (**d**) Western blot analysis of indicated cell lysates for expression of EGFR protein levels. Numbers indicate the ratio of EGFR/GAPDH for each sample. (**e**) Susceptibility of clones A and B to NK-mediated lysis at indicated E:T ratios. No lysis was observed with Clones C and D. Original magnification of all images: 20 ×. Blue corresponds to DAPI-stained nuclei. Detailed images of stained cells are shown in the insets

We hypothesized that the enhanced expression of mesenchymal proteins and, in particular, the high expression of CSC-associated markers and function in tumor cells treated with erlotinib for 72 h could be a consequence of either (1) selection of a small population of cells that previously exhibited features of EMT/CSCs in the parental cell line, or (2) induction of tumor cells into an EMT/CSC status following blockade of EGFR signaling. To investigate which of these potential mechanisms was taking place in our experimental systems, single cell-derived clonal populations of HCC827 cells were generated, expanded, and subsequently characterized for expression of E-cadherin and fibronectin by immunofluorescence (Figure 7a). Two clones each with an epithelial (A and B) or a mesenchymal-like phenotype (C and D) were selected for further study. Exposure to erlotinib revealed clear signs of apoptotic death (Figure 7b) and lower viability (Figure 7c) in clones A and B, as compared with clones C and D, suggesting that erlotinib might result on the elimination of epithelial clones, although sparing tumor cells with mesenchymal-associated features. EGFR expression levels were also assessed in the clones (Figure 7d). Interestingly, reduced levels of EGFR were observed with clones C and D, suggesting that reduced EGFR expression could be responsible for the observed responses to erlotinib. In addition, epithelial clones A and B were susceptible to NK lysis, whereas no lysis was observed with mesenchymal-like clones C and D (Figure 7e). These data, coupled with the resistance to treatment associated with features of EMT, emphasizes the necessity for a short-term time course of erlotinib treatment if combinations with immunotherapies are being conducted.

### Alleviation of resistance via IL-8 signaling blockade

**Figure 8:**
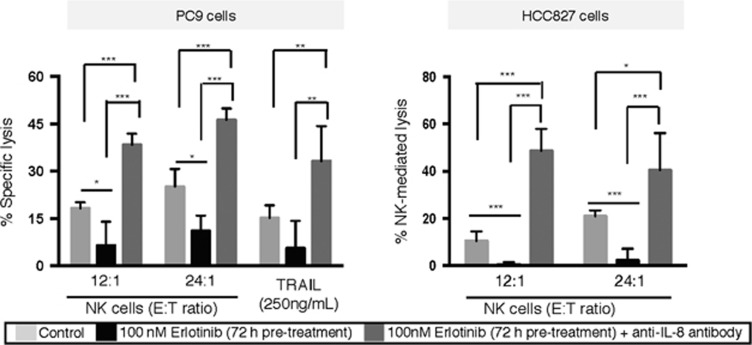
Alleviation of resistance via blockade of IL-8 signaling. Cytotoxicity of PC9 and HCC827 cells mediated by NK cells or TRAIL, as indicated. Tumor cells were left untreated, treated with erlotinib for 3 days, or treated with erlotinib for 3 days followed by exposure to anti-IL-8 neutralizing antibody before the assay

Previous work from our laboratory^27, 38^ and others^39^ have demonstrated a role for the inflammatory chemokine IL-8 in the context of acquired resistance to erlotinib. Here, we have evaluated the effect of IL-8 blockade with PC9 and HCC827 cells exposed to erlotinib for 72 h and subsequently treated with a commercially available, neutralizing anti-IL-8 antibody. As shown in Figure 8, IL-8 blockade following 72-hour erlotinib treatment enhanced tumor lysis mediated by NK effector cells or TRAIL above the levels observed with untreated tumor cells.

## Discussion

The EGFR-TKI erlotinib has been approved for years as a first-line therapy for patients harboring EGFR-sensitizing mutations. With the recent, successful implementation of immunotherapeutic strategies for the treatment of lung cancer, there is a growing interest in developing novel combinatorial therapies that could utilize immune approaches in the context of conventional or targeted therapies. In the current study, we have exploited the interconnected relationship between the EGF/EGFR axis with the induction of EMT^20^ and the ability of erlotinib to modulate the phenotype of lung cancer cell lines towards a more epithelial one. We have also implicated the role of EMT status in tumor susceptibility to immune-mediated attack, and demonstrated that treatment with erlotinib for a short period of time (16 h) promotes a general enhancement of cytotoxicity in response to NK cells, antigen-specific T cells, or TRAIL in each of the cell lines evaluated. These results are in agreement with previous reports demonstrating that erlotinib can enhance NK-mediated lysis^40^ and TRAIL-mediated apoptosis^41^ in NSCLC cells. Analysis of the potential mechanisms involved in the enhanced lysis demonstrated a role for the reversion of tumor phenotype and increased susceptibility to caspase-mediated apoptosis. In agreement with a general enhancement of apoptosis, short-term erlotinib treatment also increased tumor susceptibility to chemotherapy. The acquisition of epithelial features has been previously shown by our laboratory to favor caspase-mediated lysis of tumor cells and thus to improve cytotoxicity mediated by T cells and NK cells.^18^ Unlike epithelial tumor cells that can be efficiently lysed via caspase-dependent or independent mechanisms, we have previously shown that carcinoma cells undergoing EMT are poorly lysed by caspase-dependent mechanisms due to a defective nuclear lamin degradation as a result of decreased phosphorylation following apoptotic triggering. This apoptotic defect, however, can be overcome by perforin/granzyme-mediated pathways, as granzymes have been previously shown to directly mediate the degradation of the nuclear lamins regardless of their phosphorylation status.^42^ Our data with short-term erlotinib further demonstrates that alleviation of mesenchymal features sensitizes tumor cells to lysis by restoring susceptibility to caspase-dependent pathways.

To our knowledge, this is the first study to describe a rapid and dynamic effect of erlotinib treatment on tumor phenotype both *in vitro* and *in vivo*, and to describe how this modulation of phenotype can impact tumor sensitivity to immune attack. Our data also have implications for understanding the extensive impact of EGFR signaling in lung cancer, as the EGF/EGFR axis has been described as a mediator of resistance to pharmacotherapy *in vitro*^43^ and in patients,^44^ as an inducer of populations with CSC features,^45^ and as demonstrated here, as a rapid and dynamic modulator of tumor phenotype and tumor susceptibility to immune attack. The myriad of manners in which EGFR signaling regulates tumor cell response to treatments, combined with erlotinib being readily available in the clinic, substantiates its continued relevance as a promising therapeutic option. However, the results from this study pinpoint the dynamic and rapid shift of tumor phenotype and sensitivity to therapies and highlight the small window in which EGFR blockade can be most beneficial in the setting of combinations with immune-mediated anti-cancer approaches. Previous work from our laboratory^27, 38^ and others^39^ have shown a central role for IL-8 signaling in the context of acquired resistance to erlotinib. Here, we have extended those observations to show that IL-8 blockade following 3-day erlotinib treatment can prevent the loss of tumor lysis in response to immune effector cells. These results suggest that the use of a clinical approach capable of blocking IL-8 signaling in tumor cells may be able to overcome resistance induced by long-term exposure to erlotinib treatment.

Recent advances in the field of cancer immunotherapy have revolutionized the clinical management of lung cancer.^46, 47^ In addition to checkpoint inhibitors that eliminate the breaks on the immune system imposed by tumor cells, the use of cancer vaccines targeting tumor-associated antigens has also become an attractive therapeutic modality in recent years. Cancer vaccination as a therapeutic option stimulates the immune system, resulting in the production of antigen-specific cytotoxic T lymphocytes targeting appropriate tumor-associated antigens.^48^ In particular, our laboratory has previously developed cancer vaccine platforms aimed at targeting the transcription factor brachyury, a driver of tumor EMT^49, 50^ that is overexpressed in lung carcinomas^51, 52^ and associates with poor prognosis in patients.^53, 54, 55^ Two such vaccines are already undergoing phase I or II clinical evaluation.^56, 57^ However, vaccines as a monotherapy have shown thus far minimal tumor control and there is growing evidence that anti-tumor vaccine approaches might be most effective in patients with relatively low-tumor burden, such as following tumor debulking treatments.^58^ Taking this into consideration, erlotinib is an excellent therapy for debulking as it is associated with a marked therapeutic response in the majority of lung cancer patients with EGFR-mutated tumors. The data presented here thus provide rationale for potential combinations of erlotinib and immunotherapies for the treatment of lung carcinomas in the early setting, before the establishment of tumor relapse with long-term treatment with an EGFR-TKI.

## Materials and Methods

### Tumor cell lines and tissue culture

The HCC827 and HCC2935 human lung carcinoma cell lines were obtained from the American Type Culture Collection (ATCC) and propagated as recommended. HCC4006, H3255 and PC9 cell lines were kindly provided by Dr. Udayan Guha, NCI, NIH, Bethesda, MD, USA. Identity of the HCC827, HCC4006 and PC9 cell lines was verified by short-tandem repeat (STR) profile analysis (Biosynthesis, Lewisville, TX, USA).

### Immune effector cells

Peripheral blood mononuclear cells from healthy donors and cancer patients was obtained under the appropriate Institutional Review Board approval and informed consent. NK cells were isolated from healthy donor peripheral blood mononuclear cells by using a magnetic NK Cell Isolation Kit (Miltenyi Biotech, San Diego, CA, USA) and cultured in RPMI media containing 10% fetal bovine serum. Antigen-specific, HLA-A02 and HLA-A24-restricted cytotoxic T lymphocytes directed against an epitope of brachyury (WLLPGTSTV) or mucin-1 (MUC-1, KYHPMSEYAL), respectively, were generated and expanded as previously described from the blood of cancer patients.^59, 60^

### Cytotoxicity assay

Target cells were labeled with 20 *μ*Ci of ^111^Indium-oxine (GE Healthcare, Arlington Heights, IL, USA) for 15 min at room temperature, washed and subsequently plated at 2–3 × 10^3^ cells per well in 96-well rounded-bottom culture plates. Target cells were co-cultured with isolated NK cells at indicated effector to target (E:T) ratios, HLA-A02 brachyury-specific or HLA-A24 MUC1-specific CD8^+^ T cells (at 50:1 E:T ratio). When target cells were simultaneously treated with erlotinib, 100 nM erlotinib was added directly to the cytotoxicity assay. In the case of erlotinib pre-treated cells, target cells were incubated with erlotinib for 72 h in culture, washed, labeled with ^111^Indium and used as targets. TRAIL was used at 500 ng/ml. After an overnight incubation at 37 °C, supernatants were collected and the ^111^In released was measured by gamma counting. Spontaneous release was determined by incubating the target cells with medium alone, and complete lysis was determined by incubation of target cells with 2.5% Triton X-100. Erlotinib spontaneous release was determined by incubating target cells with medium containing 100 nM erlotinib alone. All determinations were done in at least triplicate. Specific lysis was calculated as follows: specific lysis (%)=[(observed release-spontaneous release)/(complete release−spontaneous release)] × 100.

### Erlotinib and chemotherapy response

For evaluation of responses to chemotherapy, tumor cells were either left untreated or exposed to erlotinib for 16 or 72 h and subsequently collected and plated in 96-well plates at 900 cells per well. A mixture of chemotherapy agents (10 ng/ml cisplatin and 1 ng/ml vinorelbine) was added to the cells for 96 h, followed by cell survival evaluation with Cell Titer-Glo (Promega, Madison, WI, USA). Six-to-twelve replicates were used per condition; results are expressed as percentage lysis relative to that of untreated (no chemotherapy) cells for each group (erlotinib 0, 16, 72 h).

### Erlotinib and IL-8 blockade

Tumor cells were pre-treated for 72 h with 100 nM erlotinib as indicated above, or left untreated. A third group consisted of tumor cells treated with erlotinib for 72 h followed by treatment with a commercially available neutralizing anti-IL-8 antibody (MAB208, R&D Systems, Minneapolis, MN, USA; 10 *μ*g/ml) for 96 h. Cells were labeled and used as targets with NK effector cells or recombinant TRAIL.

### Real-time PCR

Total mRNA was prepared using the RNAeasy extraction kit (Qiagen, Valencia, CA, USA) and reverse transcribed with the Advantage RT-for-PCR kit (Clontech, Mountain View, CA, USA). The resulting cDNA (10–15 ng) was amplified in triplicate using the Gene Expression Master Mix and the following TaqMan human gene expression assays (Applied Biosystems, Foster City, CA, USA): *CDH1* (Hs00610080), *CDH2* (Hs00983062), *OCT4* (Hs00742896), *Nanog* (Hs02387400), *TRAILR-1* (Hs0026491), and *TRAILR-2* (Hs00366278). Expression of each target gene relative to GAPDH was calculated as 2^-(Ct(GAPDH)–Ct(target gene)^.

### ALDEFLUOR assay

Activity of the enzyme aldehyde dehydrogenase (ALDH) was evaluated in tumor cells treated with control (DMSO) or 100 nM erlotinib for 3 days. Cells were collected and ALDH levels were assayed with an Aldefluor Assay Kit (Stem Cell Technologies, Cambridge, MA, USA), following the manufacturer's recommendations.

### Apoptosis array

For detection of apoptosis signaling in tumor cell lysates of HCC827 and PC9, a Proteome Profiler Human Apoptosis Array Kit (R&D Systems) was used, following the manufacturer's recommendations.

### Spheroid assay

Tumor cells were treated with erlotinib at 100 nM for 3 days, collected and plated (30 000 cells per well) in 24-well ultra-low-attachment plates (Corning, Corning, NY, USA) in Methocult H4100 Base Methylcellulose Medium (Stem Cell Technologies) mixed at 2:3 ratio with Iscove's Modified Dulbecco's Medium (Corning) containing 20 ng/ml EGF, 20 ng/ml fibroblast growth factor and 5 *μ*g/ml insulin. Cells were grown for ~2 weeks until spheroids developed; spheroids were subsequently collected and plated at 5000 cells per well as indicated above. Images were taken at 20 × following 2 weeks of secondary culture.

### Western blot

Protein lysates were prepared with RIPA lysis buffer (Santa Cruz Biotechnology, Santa Cruz, CA, USA), resolved (25–100 *μ*g) on SDS-PAGE and transferred onto nitrocellulose membranes using a standard western blot protocol. Membranes were probed with primary antibodies against fibronectin, E-cadherin and N-Cadherin (BD Biosciences, San Jose, CA, USA) overnight at 4 °C. Membranes were incubated with appropriate secondary antibody conjugated with IRDye and detected by the Odyssey system (Li-COR Biotechnology, Lincoln, NE, USA). For detection of multiple apoptosis-related proteins in tumor lysates from HCC827 or PC9 cells untreated or treated for 72 h with 100 nM erlotinib, a Proteome Profiler Human Apoptosis Array Kit (R&D Systems) was used, following the manufacturer's recommendations. Signal was detected and quantified by the Odyssey system (Li-COR Biotechnology).

### Immunofluorescence and IHC

Cells cultured on glass cover slips were fixed with 3% formaldehyde, permeabilized with 0.05% Triton-X and blocked with phosphate-buffered saline (PBS) containing 10% goat serum and 1% BSA. Cover slips were incubated overnight with primary antibody dilutions (1:250) prepared in 0.2% BSA in PBS 1 ×, and subsequently washed and incubated with an Alexa Fluor-488 goat anti-mouse antibody (Invitrogen, Waltham, MA, USA) for 1 h at room temperature. Slides were then exposed to DAPI (1 *μ*g/ml) in PBS at room temperature for 5 min. Cover slips were mounted using VECTASHIELD with DAPI mounting medium (Vector Laboratories, Burlingame, CA, USA). Images were captured utilizing a Leica Fluorescent microscope. IHC analysis was carried out as previously described;^61^ antibodies used were anti-E-cadherin, anti-fibronectin (GeneTex, Irvine, CA, USA) and anti-p27 (Cell Signaling, Danvers, MA, USA).

### Tumor studies *in vivo*

To establish subcutaneous tumors, six-week old female C.B17 SCID mice (Taconic, Hudson, NY, USA) were inoculated with 4 × 10^6^ cells in 100 *μ*l of Hank's balanced salt solution admixed with Matrigel 50% (v/v). All mice were housed and maintained in microisolator cages under specific pathogen-free conditions and in accordance with the Association for Assessment and Accreditation of Laboratory Animal Care (AAALAC) guidelines. All experimental studies were carried out under approval of the NIH Intramural Animal Care and Use Committee. Erlotinib was given i.p. as previously described.^62^ Briefly, mice were injected with 12.5 mg/kg/day of erlotinib (SelleckChem, Houston, TX, USA) prepared in carboxymethyl cellulose *versus* vehicle alone for 1–4 days. Tumors were collected 24 h after the last injection, formalin-fixed, and processed for immunohistological evaluation using primary antibodies against fibronectin and E-cadherin (as above). Sections were counterstained with hematoxylin.

### Statistical methods

Data were analyzed using GraphPad Prism (GraphPad Software, La Jolla, CA, USA) utilizing the two-tailed, unpaired *t*-test with alpha=0.05. Data points in graphs represent the mean±S.D. for triplicate or more measurements (*P<0.05; **P<0.01; ***P<0.001, ****P<0.0001).

## Abbreviations

CSC: cancer stem-like cell
DAPI: 4′,6-diamidino-2-phenylindole
EGF: epidermal growth factor
EGFR: epidermal growth factor receptor
EMT: epithelial–mesenchymal transition
IHC: immunohistochemistry
NK: natural killer
NSCLC: non-small cell lung cancer
PBS: phosphate buffered saline
PCR: polymerase chain reaction
siRNA: small interfering RNA
TKI: tyrosine kinase inhibitor
TRAIL: TNF-related apoptosis-inducing ligand
