# Supplementary figures and images for: Short-term EGFR blockade enhances immune-mediated cytotoxicity of EGFR mutant lung cancer cells: rationale for combination therapies
Source: Cell Death Dis. 2016 Sep 29;7(9):e2380–. doi: 10.1038/cddis.2016.297 (PMC5059888; doi:10.1038/cddis.2016.297)

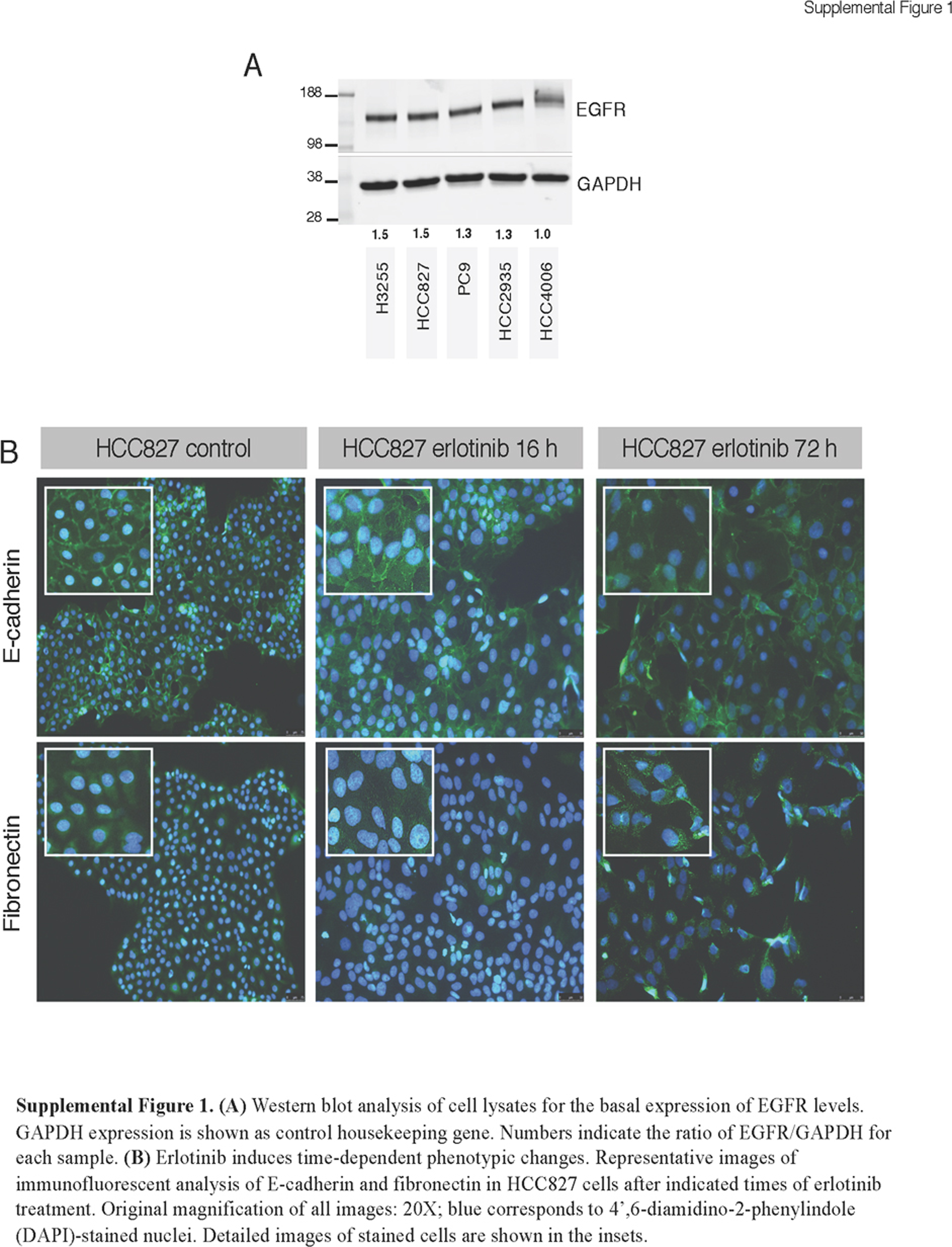

Supplement: Supplementary Figure 1 [file cddis2016297x1.tif]

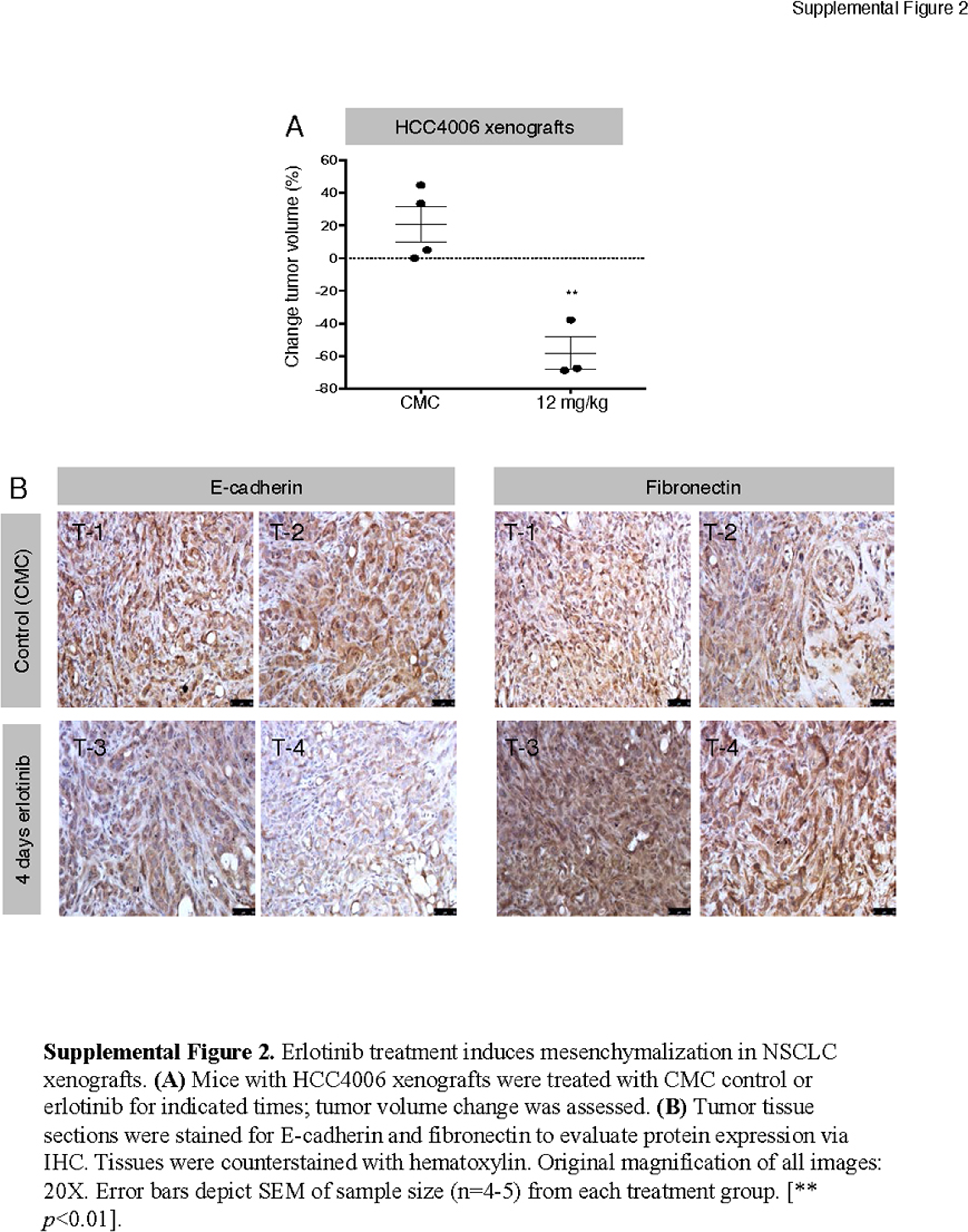

Supplement: Supplementary Figure 2 [file cddis2016297x2.tif]

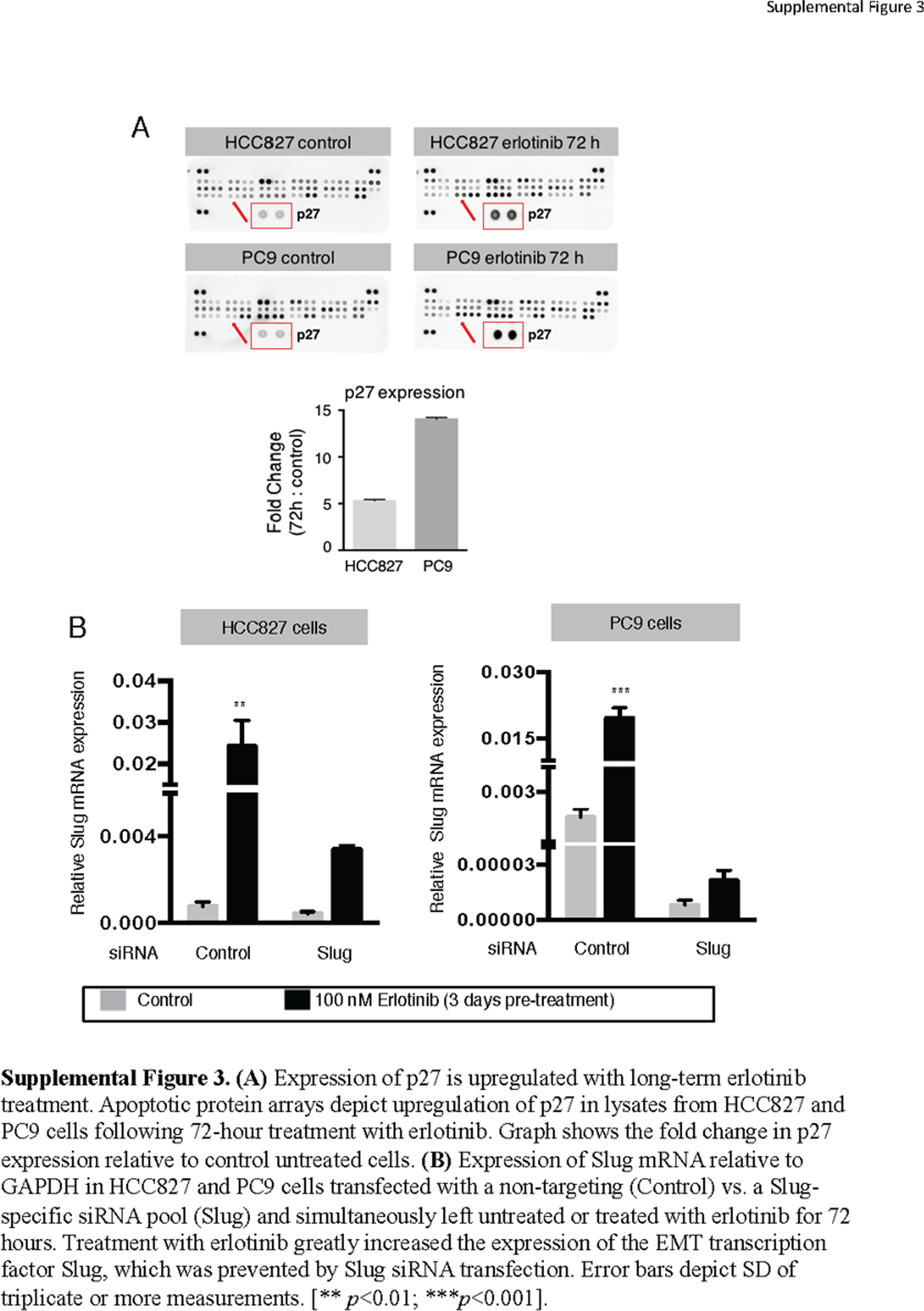

Supplement: Supplementary Figure 3 [file cddis2016297x3.tif]
